# Differences in the Volatilomic Urinary Biosignature of Prostate Cancer Patients as a Feasibility Study for the Detection of Potential Biomarkers

**DOI:** 10.3390/curroncol30050370

**Published:** 2023-05-10

**Authors:** Giulia Riccio, Cristina V. Berenguer, Rosa Perestrelo, Ferdinando Pereira, Pedro Berenguer, Cristina P. Ornelas, Ana Célia Sousa, João Aragão Vital, Maria do Carmo Pinto, Jorge A. M. Pereira, Viviana Greco, José S. Câmara

**Affiliations:** 1 Department of Basic Biotechnological Sciences, Intensivological and Perioperative Clinics, Univesità Cattolica del Sacro Cuore, 00168 Rome, Italy; 2 Unity of Chemistry, Biochemistry and Clinical Molecular Biology, Department of Diagnostic and Laboratory Medicine, Fondazione Policlinico Universitario A. Gemelli IRCCS, 00168 Rome, Italy; 3 CQM—Centro de Química da Madeira, NPRG, Universidade da Madeira, Campus da Penteada, 9020-105 Funchal, Portugalrmp@staff.uma.pt (R.P.);; 4 Serviço de Urologia, Hospital Dr. Nélio Mendonça, SESARAM, EPERAM—Serviço de Saúde da Região Autónoma da Madeira, Avenida Luís de Camões, nº57, 9004-514 Funchal, Portugal; 5 Centro de Investigação Dra Maria Isabel Mendonça, Hospital Dr. Nélio Mendonça, SESARAM, EPERAM, Avenida Luís de Camões, nº57, 9004-514 Funchal, Portugal; 6 RO-RAM—Registo Oncológico da Região Autónoma da Madeira, Hospital Dr. Nélio Mendonça, SESARAM, EPERAM, Avenida Luís de Camões, nº57, 9004-514 Funchal, Portugal; 7 Centro de Saúde do Bom Jesus, SESARAM, EPERAM, Rua das Hortas, nº67, 9050-024 Funchal, Portugal; 8 Departamento de Química, Faculdade de Ciências Exatas e Engenharia, Universidade da Madeira, Campus da Penteada, 9020-105 Funchal, Portugal

**Keywords:** prostate cancer, volatilomics, urine, biomarkers

## Abstract

Prostate cancer (PCa) continues to be the second most common malignant tumour and the main cause of oncological death in men. Investigating endogenous volatile organic metabolites (VOMs) produced by various metabolic pathways is emerging as a novel, effective, and non-invasive source of information to establish the volatilomic biosignature of PCa. In this study, headspace solid-phase microextraction combined with gas chromatography–mass spectrometry (HS-SPME/GC-MS) was used to establish the urine volatilomic profile of PCa and identify VOMs that can discriminate between the two investigated groups. This non-invasive approach was applied to oncological patients (PCa group, *n* = 26) and cancer-free individuals (control group, *n* = 30), retrieving a total of 147 VOMs from various chemical families. This included terpenes, norisoprenoid, sesquiterpenes, phenolic, sulphur and furanic compounds, ketones, alcohols, esters, aldehydes, carboxylic acid, benzene and naphthalene derivatives, hydrocarbons, and heterocyclic hydrocarbons. The data matrix was subjected to multivariate analysis, namely partial least-squares discriminant analysis (PLS-DA). Accordingly, this analysis showed that the group under study presented different volatomic profiles and suggested potential PCa biomarkers. Nevertheless, a larger cohort of samples is required to boost the predictability and accuracy of the statistical models developed.

## 1. Introduction

According to the most recent data, prostate cancer (PCa) is the second most common cancer in men and the fourth most common tumour [1]. PCa occurs mostly after 60 years old, with an average age at the time of diagnosis of 66 years old [2]. The psychological and functional states of patients are greatly impacted by PCa and following treatments, considerably affecting their quality of life [3]. The current diagnostic techniques are aggressive, costly, and uncomfortable for patients. The prostate-specific antigen (PSA) biomarker test has a low level of selectivity for diagnosing PCa and tracking cancer development [4], whereas prostate biopsies can lead to both false-positive and false-negative results [2,5,6]. Consequently, these limitations lead to overdiagnosis and overtreatment of patients [7]. Hence, there is an urgent need to identify specific and noninvasive diagnostic tools for the detection of PCa.

**Figure 1:**
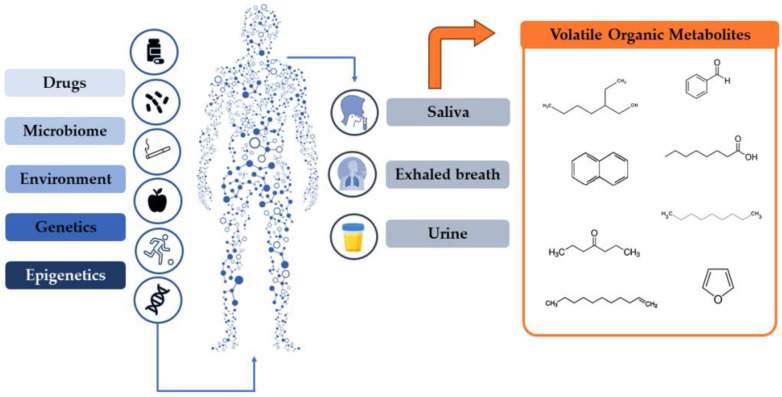
Genetic and epigenetic factors, as well as food, drugs, environment, and habits, influence the volatomic pattern in the biological fluids most used to establish the volatomic fingerprints.

Volatilomics studies volatile organic metabolites (VOMs), low-molecular-weight organic chemicals with a high vapour pressure at room temperature [8], corresponding to the volatile fraction of the metabolome [9]. VOMs are a useful source of information on the general state of health or disease status since they are produced by the metabolism of cells. Genetic, protein, and gut microbiota changes directly influence the profile of VOMs production [10]. Consequently, their production and release may be altered in some diseases, such as cancer [5,11]. Therefore, VOMs represent a patient’s metabolic fingerprint, comprising endogenous and exogenous factors, and for these reasons, have been proposed as a promising class of disease biomarkers (Figure 1) [8,12].

VOMs have been highlighted in recent studies because of their ease of use and non-invasiveness, as they can be identified in easily accessible biofluids such as urine, saliva, and exhaled breath [13,14]. VOMs contain valuable information about the biochemical metabolization of cancer cells, and each cancer type is thought to have a specific VOM pattern. Moreover, previous research has shown that VOMs can be used to distinguish between oncological and healthy individuals (Table 1) [11]. Volatilomic analysis involves sensitive analytical techniques such as mass spectrometry (MS), electronic nose (e-nose), or sensor techniques combined with multivariate statistical analysis to characterise the chemical composition of biological fluids [11,15]. MS techniques identify and quantify the levels of VOMs, whereas e-nose sensor arrays are linked to pattern recognition algorithms or chemical sensor systems [10,12].

Owing to the enrichment of volatile compounds, ranging in polarity and complexity, urine is the preferred biological fluid for volatilomic research. In addition to its reproducibility and patient acceptability, urine has fewer interfering proteins or lipids [12,34,35]. Taverna et al. [18], Filianoti et al. [19], and Capelli et al. [20] proposed different e-noses for PCa diagnosis through urinary volatilomic profiling (Table 1). The e-noses developed were able to detect alterations in the urine volatilome associated with PCa and thereby discriminated oncological patients from healthy controls, with sensitivity and specificity superior to 81% and 79%, respectively. Wen and collaborators [21] developed an extraction technique using HiSorb sorptive extraction combined with gas chromatography coupled to time-of-flight mass spectrometry (GC-TOF-MS) for urine analysis of PCa patients. The authors identified four candidate urinary biomarkers, 2-pentanone, hexanal, 3-hexanone, and *p*-cymene, which were able to discriminate patients with pancreatic ductal adenocarcinoma from non-cancer individuals. Benet et al. [22] implemented an e-nose to detect breast cancer in urine samples, which was tested using an artificial intelligence-based classification algorithm after GC-MS analysis, resulting in a sensitivity of 100% and a specificity of 50%. Exhaled breath reflects the status and condition of the metabolism. It is an acceptable approach, and its sampling is easy to use via simple hand-held devices [12,34,35]. Cheng et al. [25] proposed a prospective study consisting of the analysis of the exhaled breath of colorectal cancer patients. The samples were analysed using thermal desorption-GC-MS (TD-GC-MS), and the data were examined with machine learning techniques. The results revealed ten discriminatory VOMs in which advanced adenomas could be distinguished from negative controls with a sensitivity and specificity of 79% and 70%, respectively. Combined cancer patients and advanced adenomas could be discriminated from controls with a sensitivity and specificity of 77% and 70%, respectively. Patients with colorectal cancer were also discriminated from controls with a sensitivity of 80% and a specificity of 70%. Jung and collaborators [26] aimed to identify specific VOMs related to gastric cancer by PTR-TOF-MS. Four VOMs, propanal, aceticamide, isoprene and 1,3-propanediol, showed gradual increases as the tumour advanced, from controlled to early or advanced gastric cancer. Sukaram et al. [30] investigated the VOMs profile in the exhaled breath of hepatocellular carcinoma patients through headspace solid-phase microextraction (HS-SPME) combined with GC-MS and Support Vector Machine algorithm. A panel of six VOMs consisting of acetone, 1,4-pentadiene, methylene chloride, benzene, phenol, and allyl methyl sulfide, was correlated with the hepatocellular carcinoma stages, exhibiting an increased distance from the classification boundary when the stage advanced. Saliva collection is the easiest method for sampling biofluids. [12,34,35]. Its volatile composition reflects the oral composition, allowing relevant metabolic information [12,34,35]. Bel’skaya et al. [32] determined the volatilomic composition of saliva in stomach and colorectal cancer patients. The samples were analysed using capillary GC and showed that acetaldehyde, acetone, 2-propanol, and ethanol could discriminate between cancer and control groups with a sensitivity and specificity of 95.7 and 90.9%, respectively. Shigeyama et al. [33] established the salivary profile of patients with oral squamous cell carcinoma to investigate VOMs as potential biomarkers in the diagnosis of oral cancer. The authors combined thin-film microextraction based on a ZSM-5/polydimethylsiloxane hybrid film coupled with GC-MS and identified twelve discriminatory VOMs.

The analysis of the volatilome of PCa is still relatively recent when compared to other malignancies. Most research is based on the chemical characterisation of a biofluid or its headspace for the detection and quantification of putative PCa biomarkers through comparative analysis of samples from PCa patients and healthy controls (as reviewed by Berenguer et al. [11]). HS-SPME, developed by Arthur and Pawliszyn [36,37], combined with GC-MS, has been widely used for VOMs analysis. It is a simple, solvent-free, and sensitive extraction method that does not require a concentration step before analysis, thereby reducing the risk of interference generation [38]. Therefore, this study aimed to comprehensively characterise the urine volatilome of PCa patients by using HS-SPME/GC-MS to identify and define a set of molecular biomarkers for the diagnosis of PCa. Chromatographic data were then submitted to advanced statistical tools as a powerful way to define a pool of potential PCa biomarkers which can be used after validation for PCa diagnosis.

## 2. Materials and Methods

### 2.1. Materials and Reagents

Sodium chloride (NaCl, 99.5%) was acquired from Panreac AppliChem ITW Reagents (Barcelona, Spain) to promote salting-out of the VOMs. Ultrapure water obtained from a Milli-Q water purification system (Millipore, Bedford, PA, USA) was used to prepare the solutions hydrochloric acid (HCl, 37%) 5 M and 3-octanol (internal standard (IS), 99%) 2.5 parts per million (ppm), both from Sigma-Aldrich (St. Louis, MO, USA). For the HS-SPME procedure, the glass vials, SPME holder, and a fused silica fibre coating partially cross-linked with 50/30 µm Divinylbenzene/Carboxen/Polydimethylsiloxane (DVB/CAR/PDMS) were purchased from Supelco (Merck KGaA, Darmstadt, Germany). The DVB/CAR/PDMS fibre was used to extract a wider range of VOMs and was conditioned at 270 °C for 30 min before use, according to the manufacturer’s guidelines.

### 2.2. Subjects

A cohort of 56 men was included in this study: 30 healthy individuals without any known pathology (control group) and 26 PCa patients (PCa group) (Table 2). The control group consisted of current non-smokers with no history of prostate malignancy. These individuals also did not take any medication for age-related comorbidities or metabolic diseases such as hypertension or diabetes. Urine samples from the control group were collected during General and Family Medicine consultations at the Centro de Saúde do Bom Jesus. Urine samples from PCa patients were collected at the Urology Unit of SESARAM, EPERAM, prior to the confirmatory prostatic biopsy; therefore, before the newly diagnosed PCa patients enrolled in any kind of treatment or medication. All participants signed an informed consent form after being fully informed of the study’s objectives and protocol, which was previously approved by the local ethics committee (CES18/2022). Each urine sample was aliquoted in 8 mL vials and stored at −20 °C until analysis. All data collected from the participants were processed to ensure confidentiality, privacy, and ethical principles inherent to any research study involving human subjects.

None of the patients in this study were receiving treatment for PCa. The Urology unit follows the European Association of Urology guidelines that state that the definitive diagnosis is given by the prostatic biopsy, and no treatment should be initiated before that. Even in the cases of high-volume disease, the biopsy was taken before systemic treatment was initiated.

### 2.3. HS-SPME Procedure

HS-SPME extraction was performed according to previously optimized conditions for the analysis of the volatilomic composition of urine samples of other malignant tumours [35,39]. Briefly, 4 mL aliquots of urine sample, adjusted to pH 1–2 with 500 µL HCl (5 M), were transferred to an 8 mL sampling glass vial with 0.8 g NaCl and 5 µL 3-octanol (2.5 ppm). For the extraction of volatiles, the vial was placed in a thermostat bath adjusted to 50.0 ± 0.1 °C under stirring at 800 rpm for 60 min. After extraction, the SPME fibre was inserted into the injector port (250 °C) of the GC-MS for 6 min to desorb the analytes. The absence of 3-octanol in the samples of all studied groups was confirmed before its use as an IS.

### 2.4. GC-MS Analysis

The GC-MS analysis was performed in an Agilent Technologies 6890N Network (Palo Alto, CA, USA), equipped with a 30 m × 0.25 mm ID × 0.25 µm film thickness, BP-20 (SGE, Dortmund, Germany) fused silica column. The oven temperature was fixed at 35 °C for 2 min, increased to 220 °C (rate 2.5 °C min^−1^), and held for 5 min, for a total run time of 77 min. Helium of purity 5.0 (Air Liquide, Algés, Portugal) was used as the carrier gas at 1.1 mL min^−1^. The injection port was heated at 250 °C and operated in splitless mode. The temperatures of the transfer line, quadrupole, and ionisation source were 270 °C, 150 °C, and 230 °C, respectively. The analysis was performed in scan mode using a mass range of 30–300 m/z, and the electron impact mass spectra was 70 eV. The electron multiplier was set to auto-tune procedure, and the ionisation current was 10 mA. The identification of the VOMs was achieved by manual interpretation of the spectra and comparison with the Agilent MS ChemStation Software (Palo Alto, CA, USA), equipped with a NIST05 mass spectral library with a similarity threshold of 480%. The results are expressed as relative peak areas.

### 2.5. Statistical Analysis

MetaboAnalyst 5.0 [40] was used to perform the statistical analysis. The data matrix was normalised using a cubic root transformation and mean-centered scaling. Normalised data were processed using a *t*-test (*p*-values < 0.05). Considering the statistically significant VOMs, multivariate analysis was performed through partial least-squares discriminant analysis (PLS-DA). A heatmap using Euclidean correlation was used to identify potential clustering patterns among the significantly altered VOMs in the studied groups. The important variables of the PLS-DA model were verified according to the variable importance in projection (VIP) score and used to validate the PLS-DA models by 10-fold cross-validation (CV) and permutation tests (1000 random permutations of Y-observations).

## 3. Results and Discussion

### 3.1. Characterisation of Urinary Volatile Metabolites

**Figure 2:**
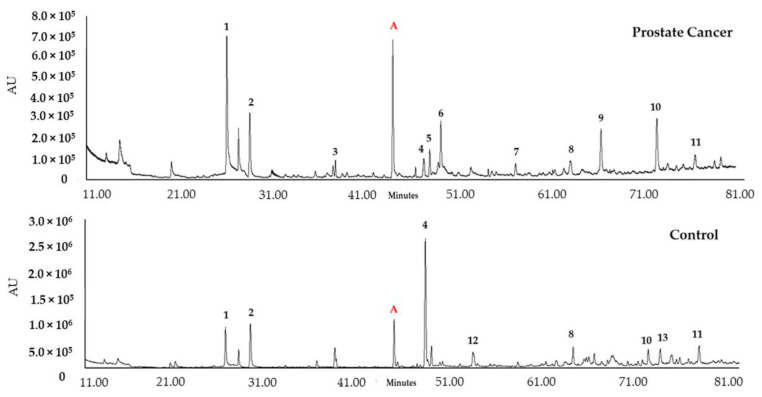
Example of typical GC-qMS urinary volatilomic profile of prostate cancer and control samples. Most important peaks: (1) Dimethyl disulfide; (2) 4-Heptanone; (3) o-Cymene; (4) p-Cymenene; (5) Dihydromyrcenol; (6) 2-Ethyl-1-hexanol; (7) Menthol; (8) D-Carvone; (9) β-Damascenone; (10) Phenol; (11) 4-Methyphenol; (12) β-Ionone; (13) 2-Bromophenol. (A) 3-Octanol, internal standard.

VOMs have been described as a promising class of biomarkers for specific diseases through the definition of volatilomic biosignatures. These sets of VOMs have the potential to be used in early detection, as diagnostic tools, and to monitor therapeutic efficacy and disease follow-up [41,42]. This study aimed to establish a urinary volatilomic profile of PCa to identify putative biomarkers for PCa diagnosis. The volatile composition of urine samples from the PCa patients (*n* = 26) and healthy subjects without any known pathology (control group, *n* = 30) (Table 1) was established using HS-SPME/GC-MS, according to the experimental procedure described. Following the HS-SPME/GC-MS analysis of the urine samples of the 56 recruited subjects, different chromatographic profiles were obtained from the control group and the PCa patients (Figure 2).

Overall, 147 VOMs were identified in the analysed samples, belonging to different chemical families, which included 13 ketones, five aldehydes, three esters, one alcohol, three carboxylic acids, seven sulfur compounds, 16 benzene derivatives, five naphthalene derivatives, 11 phenolic compounds, seven furanic compounds, 15 hydrocarbons, four heterocyclic hydrocarbons, 35 terpenes, 19 norisoprenoids, and three sesquiterpenes (Table S1, Supplementary Materials).

**Figure 3:**
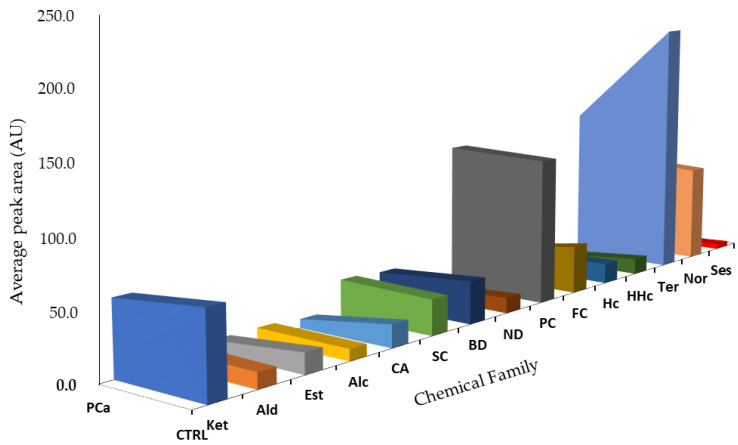
Chemical family distribution of the peak total area in the PCa (*n* = 26) and control (CTRL, *n* = 30) groups. Ket: Ketones; Ald: Aldehydes; Est: Esters; Alc: Alcohols; CA: Carboxylic Acid; SC: Sulfur Compounds; BD: Benzene Derivatives; ND: Naphthalene Derivatives; PC: Phenolic Compounds; FC: Furanic Compounds; Hc: Hydrocarbons; HHc: Heterocyclic Hydrocarbons; Ter: Terpenes; Nor: Norisoprenoids; Ses: Sesquiterpenes.

Detailed analysis of each sample group showed differences in terms of areas for the different chemical families (Figure 3). As a result of bacterial activity, metabolism, pH changes, or breakdown of urine constituents, the human urinary profile changes over time. It is also influenced by external factors, including health status, dietary habits, physical stress, and environmental exposure, which along with exogenous compounds, contribute to an individual’s volatilomic profile [11]. Due to these factors, the human metabolism is very complex, and cancer development and progression make it even more difficult to understand all the metabolic processes that may contribute to an increase or decrease in certain metabolites [35,43,44]. Thus, it is crucial to establish a relationship between the identified VOMs and their potential endogenous origin; however, the origin of many VOMs has not been clearly defined [8].

Terpenes, phenolic compounds, and norisoprenoids were the chemical families that contributed the most to the volatilomic pattern of the studied groups (Figure 3). Norisoprenoids, phenolic, and terpenic compounds can be easily found in different exogenous sources such as food [45,46]. Nevertheless, many metabolites belonging to these chemical families originate from endogenous metabolic processes in our organism, namely *p*-cymenene, *p*-cymene, 2-bromophenol, phenol, and *p*-cresol [47]. Terpenes come from the mevalonic acid pathway [35,43] and can also result from the consumption of foods and beverages [47]. 3,5-Dimethylbenzaldehyde, 2-methoxy-5-methylthiophene (MMT), 1,1,6-trimethyl-1,2-dihydronapththalene (TDN), and 2-ethyl-1-hexanol were the most abundant metabolites in the PCa group. TDN is typically found in liquorice tasting, alcoholic beverages and fruits [44,47]. 2-Ethyl-1-hexanol is a fatty alcohol in lipid molecules; it can be found in foods such as different kinds of tea, cereals and cereal products, fats and oils, and alcoholic beverages [44,47]. Furthermore, 2-ethyl-1-hexanol has been detected in five types of cancer, namely lung, laryngeal, thyroid, colorectal, and breast [8]. *o*-Cymene has been proposed as a putative biomarker of citrus ingestion since this compound is frequently found in citrus fruits [44,47].

According to the literature, ketones are one of the most abundant chemical families in the volatile profile of urine [43,48]. They are products of different metabolic pathways, namely carbohydrate metabolism and lipid oxidation processes [49,50]. A few studies have proposed that a considerable fraction of ketones in urine arises from the action of gut bacteria, but ketones can also come from exogenous sources, such as food (beverages, foods, and flavouring ingredients) or environmental pollution [8]. 2-Pentanone, the simplest ketone identified, has been found in different foods, including fruits, cereals, milk, herbs and spice, fats, and oils. Moreover, 2-pentanone has been linked to diseases such as ulcerative colitis, non-alcoholic fatty liver disease, Crohn’s disease, and also to the inborn metabolic disorder of celiac disease [47]. 4-Heptanone is one of the most common VOMs in urine; its origin is still unknown, but it may be associated with the β-oxidation of 2-ethylhexanoic acid [8]. In addition to dietary sources, 3-hexanone has been associated with several diseases, including non-alcoholic fatty liver disease, autism, and inborn metabolic disorder celiac disease [8].

Similar to ketones, sulfur compounds have been described to possess a high expression in the human urinary volatilomic profile [35,51]. Most of these metabolites are produced during the transamination pathway by the incomplete metabolism of methionine and cysteine [35,52,53,54]. During transamination, methionine and cysteine are transformed into methanethiol [55]. Then, methanethiol is easily oxidized to dimethyl disulfide and dimethyl trisulfide [53]. It has been described that Gram-negative bacteria may also produce considerable amounts of methanethiol and dimethyl disulfide [56]. Furthermore, these compounds can also result from dietary sources since dimethyl disulfide and dimethyl trisulfide are present in many foods and beverages. MMT is one of the most abundant sulfur compounds among the PCa group.

Alcohols can originate from different sources, such as the reduction of fatty acids in the gastrointestinal tract, pyruvate, citrate, or glycolysis pathways [57], or even the metabolism of hydrocarbons [8]. Similarly, the metabolism of microorganisms such as bacteria can also be a source of these metabolites [58]. Another source of alcohols is diet through the ingestion of food and beverages [8]. Dihydromyrcenol was previously detected in the urine samples of PCa patients [55] and was reported at lower levels than in control subjects [8].

Hydrocarbons are metabolites of great diagnostic interest because they are closely related to oxidative stress [59]. Alkanes and other methylated hydrocarbons typically result from the lipid peroxidation of polyunsaturated fatty acids found mainly in cell membranes [59]. Significant changes in the levels of alkanes and methyl alkanes in cancer patients may be related to the activity of CYP 450 enzymes [8]. In contrast, unsaturated hydrocarbons, typically alkenes, are often involved in the mevalonic acid pathway of cholesterol synthesis [59]. Polycyclic aromatic hydrocarbons (PAHs) are carcinogenic substances that humans are exposed to in the environment, at certain industrial workplaces, and from tobacco smoke [59]. Naphthalene is a PAH often associated with cancer development and is released by industrial, domestic, and natural burning processes, leading to exposure of the general population [59,60]. However, no metabolic pathway has clearly explained the origin of naphthalene derivatives in urine. Some researchers have indicated a potential relationship with steroid metabolism, while others have suggested that these compounds may come from the environment to which the individual is exposed [59,60].

Furanic compounds and benzene derivatives can be found in both exogenous and endogenous sources as metabolic products of food and different processes in the human organism [45,46,47]. The thermal degradation and rearrangement of carbohydrates in natural and processed food is the primary source of furanic compounds [44,46,47]. Furan was proposed as a PCa biomarker by Jiménez-Pacheco et al. [61]. 2-Methyl-5-(methylthio)furan, a furanic compound found in both the control and PCa groups, has been found in coffee, garlic, and horseradish. Benzene derivates are often related to environmental sources, such as air and environmental pollution from industrial (pesticides, dyes) or natural processes (fires). The major sources of benzene exposure are automobile service stations and tobacco smoke [48].

### 3.2. Chemometric Analysis of Urine Samples

MetaboAnalyst 5.0 [40] was used to perform the statistical analysis. The variables were initially normalised to obtain a homogeneous distribution and generate reliable and interpretable models. The normalised matrix was subjected to univariate analysis using a *t*-test (*p* < 0.05), in which the *p* values obtained proved that 7 of the 147 VOMs identified presented statistically significant differences between the analysed groups, the healthy subjects (control group), and oncological patients (PCa group) (Table 3). Some of these metabolites have been previously related to oncological pathologies, according to the Human Metabolome Database [8]. TDN has been detected in urine samples of colorectal, leukaemia, and lymphoma cancers, where it was found increasingly expressed in the samples of oncological patients [8]. About 3,5-dimethylbenzaldehyde, very little information has been published in the literature, but similar molecules, such as the isomer 2,5-dimethylbenzaldehyde or benzaldehyde, have already been related to prostate [41] and lung [62] cancers. For many VOMs related to the control group, such as D-carvone, 6-methylphenanthedrine, α-methylcinnamaldehyde, and 2-bromophenol, a significant decrease in concentration was observed in the PCa group. Although the origin of some of these metabolites is known, most of them still need more detailed evaluation to establish a relationship with PCa.

**Figure 4:**
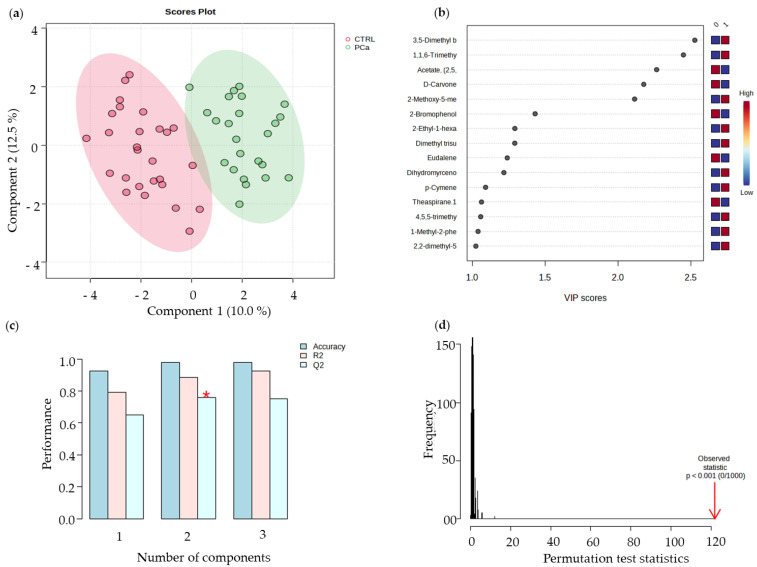
(**a**) Partial least-squares discriminant analysis (PLS-DA). (**b**) Variables of importance in projection (VIP) scores plot, representing the important features identified by the PLS-DA. The coloured boxes on the right indicate the relative concentrations of the corresponding metabolites in each group under study. (**c**) 10-fold CV performance of the PLS-DA classification using a different number of components (* means best Q^2^ value, the best classifier). (**d**) PLS-DA model validation by permutation tests based on 1000 permutations of the VOMs obtained by GC-MS of the urine samples from the groups under study.

PLS-DA multivariate pattern recognition procedures use the information contained in the VOMs fingerprint as several variables to visualize group trends and clustering patterns, respectively, according to the separations among sample sets. The resulting PLS-DA analysis showed two well-separated groups, the PCa and the control groups (Figure 4a). Besides the significant difference between PCa patients and healthy subjects (control group) in terms of smoking habits and age, these factors did not contribute to the differences noted between both groups. When carrying out the discriminant statistical analysis by age and by smoking habits, it was verified that no cluster was formed associated with any of the target groups. Hence, it can be deduced that neither age nor the difference in the number of smokers between both groups influenced the separation obtained among the PCa cluster and control group cluster. The VIP scores plot describes the relative contribution of the metabolites to the variance between the two groups, where TDN and D-carvone showed the most significant contributions to the PCa and control groups, respectively (Figure 4b). The robustness of the generated PLS-DA model was evaluated by 10-fold CV (Figure 4c), and to assess the significance of class discrimination, a permutation test was performed (Figure 4d). The resulting PLS-DA analysis showed two well-separated groups. The VIP scores plot describes the relative contribution of the metabolites to the variance between the two groups. TDN and D-carvone showed the most significant contributions to the PCa and control groups, respectively.

**Figure 5:**
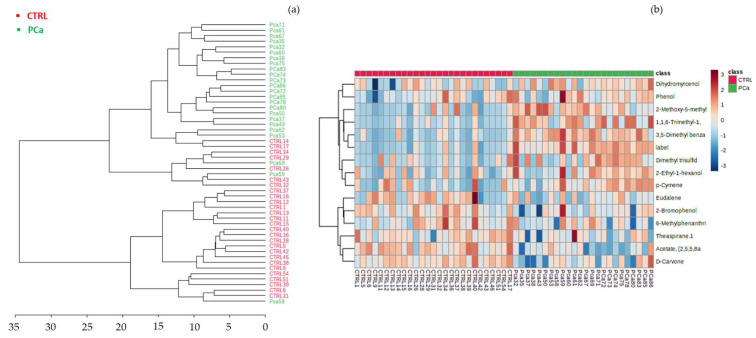
Hierarchical cluster analysis of CTRL (control) and Pca (prostate cancer) groups (**a**) Dendrogram analysis of the volatomic data, using Euclidean distance measure and Ward’s linkage. (**b**) Clustering result shown as heatmap illustrates the concentration of the urinary volatile organic metabolites identified in each sample. Columns correspond to Pca and CTRL sample groups, respectively, whereas rows correspond to the most relevant VOMs detected. The colour of the cells corresponds to the normalised peak areas of the compounds (minimum −1, dark blue; maximum +1, dark red).

Hierarchical clustering was performed, resulting in a dendrogram and heatmap (Figure 5). The heatmap created using Euclidean distance measure with the 15 statistically significant VOMs illustrated the correlations between these VOMs and the sample groups (Figure 5b). This hierarchical cluster analysis showed that each cluster of the studied groups was well-defined by a distinct panel of metabolites. For instance, D-carvone, *p*-cymenene, and 2-bromophenol *p*-tert-butylphenol were the metabolites most associated with the control group, whereas 3,5-dimethylbenzaldehyde, MMT, TDN, and 2-ethyl-1-hexanol were highly correlated with the PCa group.

**Figure 6:**
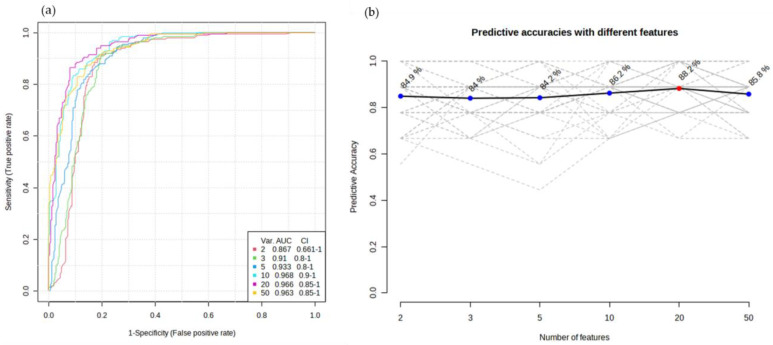
(**a**) ROC curves for the most important features with the highest ability to discriminate both groups. (**b**) Plot of the predictive accuracy of biomarker models with an increasing number of features. The most accurate biomarker model is highlighted with a red dot.

To evaluate the performance of the potential biomarker models, the multivariate exploratory receiver operating characteristic (ROC) curves were generated by Monte Carlo cross-validation (MCCV), using 2/3 of the samples to evaluate feature importance, and the remaining 1/3 were used to validate the created models (Figure 6a,b). The top-ranking features in terms of importance were used to build the classification models. Figure 6a shows the ROC curves of a set of six volatiles based on the average cross-validation performance. The obtained values for the area under the curves (AUC) between 0.867 and 0.968, with a 95% confidence interval, are excellent and represent a good accuracy in discriminating both groups. Figure 6b shows the plot of the predictive accuracy of biomarker models with an increasing number of features.

**Figure 7:**
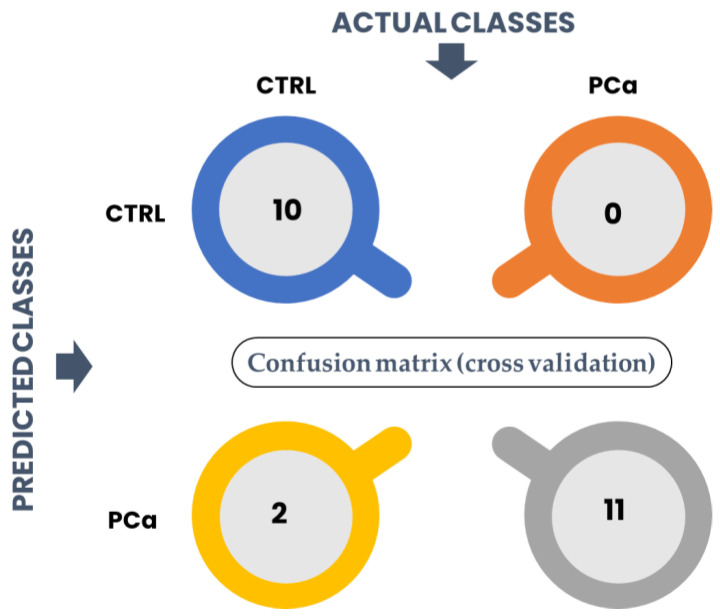
Structure of a 2 × 2 confusion matrix to assess the performance of a classification model.

The performance of the classification model was assessed through a confusion matrix was performed based on the classification method: PLS-DA. The columns represent the actual classes the outcomes should have been, while the rows represent the predictions we have made. The number of correct and incorrect predictions is summarized in Figure 7.

Our model predicted that 10/12 were from control groups when there were 12/12. The accuracy corresponds to the proportion of predictions that the model classified correctly. In this case, the accuracy of the model was 91.3% as it predicted that two healthy individuals belong to the PCa group (two false positives). The precision of the model, related to the proportion of positive identifications that were correct, was 85%. The sensitivity which expresses the proportion of actual positives identified correctly was 100%, whereas the specificity associated with the proportion of actual negatives that are correctly identified was 83.3%.

## 4. Conclusions

A total of 147 VOMs were identified as belonging to different chemical families, and different chromatographic profiles were retrieved for the groups of subjects recruited. Terpenes, phenolic compounds, and norisoprenoids were the chemical families that contributed the most to the volatilomic profile of the three studied groups: control and PCa. The statistical analysis revealed that 7 of the 147 VOMs identified presented statistically significant differences between the recruited groups, according to the *t*-test (*p* < 0.05). PLS-DA was performed, and the robustness of the generated model was evaluated using 10-fold CV and permutation tests. PLS-DA showed two well-separated groups, and the VIP score showed the most relevant metabolites among the studied groups. Hierarchical cluster analysis, carried out by Euclidean distance measure and Ward’s linkage, showed that each cluster of the studied groups was well defined by a distinct panel of metabolites. The metabolites D-carvone, *p*-cymenene, 2-bromophenol, and *p*-tert-butylphenol were more strongly associated with the control group, whereas 3,5-dimethylbenzaldehyde, MMT, TDN, and 2-ethyl-1-hexanol were highly correlated with the PCa group. A significant increase in the peak area of TDN and 3,5-dimethylbenzaldehyde was observed in PCa patients. On average, significantly lower abundances of D-carvone, 6-methylphenanthridine, α-methylcinnamaldehyde, 2-bromophenol, and 2,5,5,8a-tetramethyl-1,2,3,5,6,7,8,8-octahydro-1-naphthalenyl ester acetate (TONEA) were found in cancer patients. Further validation of the findings in this study is required using a much larger sample cohort to improve the predictive power and reliability of the developed statistical models. Likewise, additional research is required to determine which of the metabolites are of endogenous origin, disease-related, and which originate from exogenous sources, related to normal metabolic processes and external contaminations (environment or diet).

## Figures and Tables

**Table 1:** Recent studies on volatile organic metabolites for the identification of cancer biomarkers found in urine, exhaled breath, and saliva.

Cancer Type	Analytical Approach	Biomarker’s Candidates/ Findings	Prediction Model	Validation Characteristics	Reference
		Urine			
Pancreatic	TD-GC-TOF-MS GC-IMS	2,6-Dimethyl-octane, nonanal, 4-ethyl-1,2-dimethyl-benzene, 2-pentanone	Repeated 10-Fold CV	NA	[16]
Bladder, prostate	GC-TOF-MS and GC-IMS	35 VOMs	ROC, Repeated 10-Fold CV	GC-IMS Sens: 87% Spec: 92% AUC: 0.95	[17]
Prostate	Urine HS conditioning, followed by e-nose analysis	The e-nose detected alterations in the urine volatilome associated with PCa	ROC	Sens: 85% Spec: 79% AUC: 0.82	[18]
Prostate	Urine HS conditioning, followed by e-nose analysis (Cyranose C320)	The e-nose discriminated the urine smell prints of patients with PCa from healthy controls	PCA, ROC	Sens: 83% Spec: 88% AUC: 0.90	[19]
Prostate	Urine HS conditioning, followed by e-nose analysis	The e-nose discriminated patients with PCa from healthy controls	PCA	Sens: 82% Spec: 87% AUC: NA	[20]
Pancreatic ductal adenocarcinoma	HiSorb probes coupled with GC-TOF-MS	2-Pentanone, hexanal, 3-hexanone, *p*-cymene	PLS-DA	AUC: 0.82 CER: 0.18	[21]
Breast	GC-MS analysis of the urine HS. Sample’s smell print by the e-nose prototype	The e-nose software discriminated between early stage breast cancer and healthy controls	Artificial intelligence-based algorithm: CNN	Sens: 100% Spec: 50% Classification rate: 75%	[22]
Bladder	HS-SPME/GCxGC TOF-MS	Butyrolactone, 2-methoxyphenol, 3-methoxy-5-methylphenol, 1-(2,6,6-trimethylcyclohexa-1,3-dien-1-yl)-2-buten-1-one, nootkatone, 1-(2,6,6-trimethyl-1-cyclohexenyl)-2-buten-1-one	ANN	NA	[23]
Lung	GC-IMS	2-Pentanone, 2-hexenal, 2-hexen-1-ol, hept-4-en-2-ol, 2-heptanone, 3-octen-2-one, 4-methylpentanol, 4-methyl-octane	SVM	GC-IMS Sens: 85% Spec: 90% AUC: 0.91	[24]
		Exhaled breath			
Colorectal	Thermal desorption-GC-TOF-MS	10 VOMs distinguished advanced adenomas from negative controls. Colorectal cancer patients and advanced adenoma combined were discriminated from controls	RF	Colorectal cancer vs. controls Sens: 80% Spec: 70%	[25]
Gastric	PTR-TOF-MS	Propanal, aceticamide, isoprene, 1,3-propanediol	ROC	Sens: 61% Spec: 94% AUC: 0.842	[26]
Breast	SIFT-MS	3,7-Dimethyl-2,6-octadien-1-ol, ethanolamine, ethyl nonanoate	PCA, MLR	Sens: 86.3% Spec: 55.6%	[27]
Hepatocellular	SPME/GC-MS	Phenol 2,2 methylene bis [6-(1,1-dimethyl ethyl)-4-methyl] (MBMBP)	PCA	NA	[28]
Lung	HPPI-TOFMS	Isoprene, hexanal, pentanal, propylcyclohexane, nonanal, 2,2-dimethyldecane, heptanal, decanal	Hosmer–Lemeshow test	Sens: 86% Spec: 87.2% Acc: 86.9% AUC: 0.931	[29]
Hepatocellular carcinoma	HS-SPME/GC-MS	Acetone, 1,4-pentadiene, methylene chloride, benzene, phenol, allyl methyl sulfide	SVM	Sens: 44% Spec: 75% Acc: 55.4%	[30]
		Saliva			
Oral	HS-SPME/GC-MS	1-Octen-3-ol, hexanoic acid, E-2-octenal, heptanoic acid, octanoic acid, E-2-nonenal, nonanoic acid, 2,4-decadienal, 9-undecenoic acid	PCA	Sens: 100% Spec: 100% AUC: 1	[31]
Stomach and colorectal cancer	Capillary GC-FID	Acetaldehyde, acetone, 2-propanol, ethanol	CART	Sens: 95.7% Spec: 90.9%	[32]
Oral squamous cell carcinoma	Thin-film microextraction based on a ZSM-5/polydimethylsiloxane hybrid film coupled with GC-MS	12 VOMs	PCA	Sens: 95.8% Spec: 94%	[33]

Legend: Acc: accuracy; ANN: artificial neural networks; AUC: area under the receiver operating characteristic (ROC) curve; CART: classification and regression tree; CER: classification error rate; CNN: convolutional neural network; CV: cross-validation; GC-IMS: gas chromatography–ion migration spectroscopy; GC-MS: gas chromatography–mass spectrometry; GC-TOF-MS: gas chromatography coupled to time-of-flight mass spectrometry; HPPI-TOFMS: high-pressure photon ionization time-of-flight mass spectrometry; HS: headspace; HS-SPME: headspace solid-phase microextraction; MLR: multiple logistic regression; NA: not analyzed; PCa: prostate cancer; PCA: principal component analysis; PLS-DA: partial least-squares discriminant analysis; PTR-TOF-MS: proton-transfer-reaction time-of-flight mass spectrometry; RF: random forest; ROC: receiver operating characteristic; Sens: sensitivity; SIFT-MS: selected ion flow tube–mass spectrometry; Spec: specificity; SVM: support vector machine; TD-GC-MS: thermal desorption gas chromatography–mass spectrometry; TD-GC-TOF-MS: two-dimensional gas chromatography with time-of-flight mass spectrometer.

**Table 2:** Demographic and clinical data of the cancer-free controls and prostate cancer patients included in this study.

Characteristics	Control	Prostate Cancer
**Number of subjects**	30	26
**Mean age ± SD (years)**	46.21 ± 11.58	66.92 ± 9.14
**BMI (kg/m^2^) ± SD**	27.67 ± 3.78	27.34 ± 3.40
**Smoking habits**		
Ever smokers	6	16
Never smokers	19	10
Unknown	5	0
**PSA (ng/mL), *n* (%)**		
<4	30 (100%)	1 (3.85%)
4–10	-	13 (50.00%)
>10	-	12 (46.15%)
**Gleason score, *n* (%)**		
≤6	-	4 (15.38%)
7	-	12 (46.15%)
≥8	-	10 (38.46%)
**Grade group, *n* (%)**		
1	-	4 (15.38%)
2	-	8 (30.77%)
3	-	4 (15.38%)
4	-	9 (34.62%)
5	-	1 (3.85%)

Legend: BMI: body mass index; SD: standard deviation.

**Table 3:** Important features identified using the *t*-tests.

No.	Significant VOMs	*t*-Stat	*p*-Value	=−LOG10(p)	FDR
1	3,5-Dimethylbenzaldehyde	−7.479	6.87 × 10^−10^	9.1628	3.92 × 10^−8^
2	TDN	−5.7798	3.84 × 10^−7^	6.4162	1.09 × 10^−5^
3	D-Carvone	4.363	5.82 × 10^−5^	4.235	0.001106
4	6-Methylphenanthridine	3.8847	0.000282	3.55	0.004016
5	α-Methylcinnamaldehyde	3.6128	0.000665	3.1772	0.007581
6	2-Bromophenol	3.486	0.000982	3.0079	0.009328
7	TONEA	3.3169	0.001633	2.7871	0.013294

Abbreviations: TDN: 1,1,6-Trimethyl-1,2-dihydronaphthalene; TONEA: 2,5,5,8a-tetramethyl-1,2,3,5,6,7,8,8-octahydro-1-naphthalenyl ester acetate; FDR: false discovery rate.

## Data Availability

Not applicable.

## Supplementary Materials

The following supporting information can be downloaded at: https://www.mdpi.com/article/10.3390/curroncol30050370/s1, Table S1. Identified metabolites in urine samples of PCa patients and healthy subjects. Retention time (RT) in min, formula, CAS number and chemical family is reported for each compound. Frequency of occurrence as a percentage (%) and mean relative peak areas are reported for prostate cancer and control groups (n = 3; RSD <20%).
