# Supplementary material for: Differences in the Volatilomic Urinary Biosignature of Prostate Cancer Patients as a Feasibility Study for the Detection of Potential Biomarkers
Source: Curr Oncol. 2023 May 10;30(5):4904–21. doi: 10.3390/curroncol30050370 (PMC10217291; doi:10.3390/curroncol30050370)
Supplement: Supplementary file 1 [file curroncol-30-00370-s001.zip › curroncol-2286897-supplementary.pdf]

Table S1. Identified metabolites in urine samples of PCa patients and healthy subjects. Retention time (RT) in min, formula, CAS number and chemical family is reported for each compound. Frequency of occurrence as a percentage (%) and mean relative peak areas are reported for prostate cancer and control groups (*n* = 3; RSD < 20 %).

RT (min)	VOM	Formula	CAS No.	Chemical family	Frequency of occurrence (%)		Mean relative peak area	
					PCa	CTRL	PCa	CTRL
20.13	6-Methylphenanthridine	$C_{14}H_{11}N$	3955-65-5	HHc	81	100	4.38	10.50
20.77	2-Pentanone	$C_5H_{10}O$	107-87-9	Ket	54	57	3.65	5.70
22.30	3-Nonyne	C_9H_{16}	20184-89-8	Hc	4	3	0.24	0.09
22.93	1-Methylcycloheptene	C_8H_{14}	55308-20-8	Hc	19	10	1.22	0.92
23.37	(+)-Linalool	$C_{10}H_{18}$	10281-56-8	Ter	4	0	0.26	-
23.61	1,3-Dimethyl-1-cyclohexene	C_8H_{14}	2808-76-6	Hc	50	37	3.09	3.04
23.90	1-Decene	$C_{10}H_{20}$	872-05-9	Hc	4	0	0.17	-
24.41	3-Hexanone	$C_6H_{12}O$	589-38-8	Ket	19	13	0.66	0.39
25.23	*p*-Menth-3-ene	$C_{10}H_{18}$	500-00-5	Ter	4	7	0.80	0.30
26.14	Dimethyl disulfide	$C_2H_6S_2$	624-92-0	SC	100	100	20.01	20.88
26.39	Hexanal	$C_6H_{12}O$	66-25-1	Ald	19	20	1.92	1.36
28.64	4-Heptanone	$C_7H_{14}O$	123-19-3	Ket	100	100	30.76	37.48
29.85	*o*-Xylene	C_8H_{10}	95-47-6	BD	4	3	0.14	0.10
31.89	1,4-Cineol	$C_{10}H_{18}O$	470-67-7	Ter	4	13	0.24	0.57
31.96	2-Carene	$C_{10}H_{16}$	554-61-0	Ter	4	20	0.15	2.23
32.06	α-Terpinene	$C_{10}H_{16}$	99-86-5	Ter	19	23	1.23	2.00
32.58	2-Heptanone	$C_7H_{14}O$	110-43-0	Ket	4	0	0.14	-
33.86	4-Methyl-2-heptanone	$C_8H_{16}O$	6137-06-0	Ket	8	0	0.15	-
33.99	Eucalyptol	$C_{10}H_{18}O$	470-82-6	Ter	4	10	0.10	1.09
34.70	1-Pentylcyclohexene	$C_{11}H_{20}$	15232-85-6	Hc	0	7	-	0.84
35.68	2,2-dimethyl-5-(1-methyl-1-propenyl)-tetrahydrofuran	$C_{10}H_{18}O$	7416-35-5	FC	96	83	10.01	11.90
36.18	γ-Terpinene	$C_{10}H_{16}$	99-85-4	Ter	15	20	0.36	1.32
37.04	3,5-Dihydroxybenzamide	$C_7H_7NO_3$	3147-62-4	BD	15	3	1.27	0.08
37.09	3,4-Dimethylthiophene	C_6H_8S	632-15-5	SC	4	10	0.08	0.42

37.59	*p*-Cymene	$C_{10}H_{14}$	99-87-6	Ter	81	33	12.52	14.86
37.85	*o*-Cymene	$C_{10}H_{14}$	527-84-4	Ter	58	67	4.91	25.73
38.54	α-Terpinene	$C_{10}H_{16}$	99-86-5	Ter	15	7	1.97	0.74
38.62	*m*-Cymene	$C_{10}H_{14}$	535-77-3	Ter	4	3	0.06	0.06
38.79	Allyl methyl disulfide	$C_4H_8S_2$	2179-58-0	SC	8	17	0.86	1.32
39.05	Isoterpinolene	$C_{10}H_{16}$	586-63-0	Ter	35	3	1.37	0.69
40.13	1,4,6-Trimethylnaphthalene	$C_{13}H_{14}$	2131-42-2	Nor	8	0	0.85	-
40.41	1,6,7-Trimethylnaphthalene	$C_{13}H_{14}$	2245-38-7	Nor	4	10	0.32	0.55
40.88	2,6-Dimethyl-1,6-octadiene	$C_{10}H_{18}$	80-49-35	Hc	4	0	0.28	-
40.97	1,3-Cycloheptadiene	C_7H_{10}	4054-38-0	Hc	12	3	0.87	0.12
40.97	2,3,6-Trimethylnaphthalene	$C_{13}H_{14}$	829-26-5	Nor	4	0	0.23	-
40.98	2,2,6-Trimethylcyclohexanone	$C_9H_{16}O$	2408-37-9	Ket	4	10	0.14	0.28
41.97	Hemimellitene	C_9H_{12}	526-73-8	BD	15	3	0.95	0.26
42.10	*p*-Ethyltoluene	C_9H_{12}	622-96-8	BD	8	20	0.85	2.10
43.15	*p*-Methylstyrene	C_9H_{10}	622-97-9	BD	4	7	0.05	0.23
44.37	2-Methoxy-5-methylthiophene	C_6H_8OS	31053-55-1	SC	46	13	4.29	1.13
44.47	2-Methyl-5-(methylthio)furan	C_6H_8OS	13678-59-6	FC	42	73	5.32	6.12
44.58	Nonanal	$C_9H_{18}O$	124-19-6	Ald	12	0	1.73	-
44.95	Dimethyl trisulfide	$C_2H_6S_3$	3658-80-8	SC	46	17	2.79	1.23
45.19	2-Carene	$C_{10}H_{16}$	554-61-0	Ter	0	3	-	0.12
45.21	Allo-Ocimene	$C_{10}H_{16}$	673-84-7	Ter	0	7	-	0.28
46.09	Tetrahydrideinalool	$C_{10}H_{22}O$	57706-88-4	Ter	19	20	1.90	3.84
46.36	*m*-Di-tert-butylbenzene	$C_{14}H_{22}$	1014-60-4	BD	0	3	-	0.27
46.88	1,5,7-trimethyl-1,2,3,4-tetrahydronaphthalene	$C_{13}H_{18}$	21693-55-0	Nor	0	3	-	0.37
47.33	*p*-Cymenene	$C_{10}H_{12}$	1195-32-0	Ter	96	97	19.08	39.30
47.64	*o*-Cymenene	$C_{10}H_{12}$	7399-49-7	Ter	0	10	-	0.66
47.69	3-Methyl-2-butenoic acid, tridec-2-ynyl ester	$C_{18}H_{30}O_2$	100029-93-1	Est	4	0	0.53	0.00
48.04	Dihydromyrcenol	$C_{10}H_{20}O$	18479-58-8	Ter	96	83	17.57	11.58
48.11	α-Ionene	$C_{13}H_{18}$	30316-36-0	Nor	0	7	-	1.11

48.95	*t*-Furan linalool oxide	$C_{10}H_{18}O_2$	34995-77-2	FC	46	57	2.31	6.72
49.05	Nerol oxide	$C_{10}H_{16}O$	1786-08-9	Ter	4	0	0.78	-
49.23	2-Ethyl-1-hexanol	$C_8H_{18}O$	104-76-7	Alc	100	100	9.19	8.92
49.42	2-Mercapto-4-phenylthiazole	$C_9H_7NS_2$	2103-88-0	HHc	12	0	1.65	0.00
50.52	Prehnitene	$C_{10}H_{14}$	488-23-3	Ter	58	50	5.29	4.83
51.04	Theaspirane	$C_{13}H_{22}O$	36431-72-8	Nor	69	67	3.99	6.30
51.57	Carvestrene	$C_{10}H_{16}$	499-03-6	Ter	4	0	0.20	-
52.25	2,5-Diisopropyl-p-xylene	$C_{14}H_{22}$	10375-96-9	BD	0	13	-	0.98
52.46	β-Ionone	$C_{13}H_{20}O$	6901-97-9	Nor	85	83	22.00	28.77
52.92	Theaspirane	$C_{13}H_{22}O$	100019-43-4	No	65	80	3.85	7.31
53.18	2-(Methylthio)thiophene	$C_5H_6S_2$	5780-36-9	SC	8	0	0.26	-
53.43	Cadalene	$C_{15}H_{18}$	483-78-3	Ses	8	10	0.17	0.80
54.10	α-Ionene	$C_{13}H_{18}$	475-03-6	Nor	12	27	0.93	1.15
54.70	5-Methylfurfural	$C_6H_6O_2$	620-02-0	FC	42	40	4.56	3.83
54.70	2-Fenchanol	$C_{10}H_{18}O$	1632-73-1	Ter	8	0	0.20	-
54.84	1-Methylene-spiro[4.4]nonane	$C_{10}H_{16}$	19144-06-0	Hc	0	10	-	0.55
55.20	(±)-Neoisomenthol	$C_{10}H_{20}O$	491-02-1	Ter	54	57	5.66	4.66
55.79	Terpinene-4-ol	$C_{10}H_{18}O$	000562-74-3	Ter	8	7	0.14	1.12
55.98	2-Methyl-benzofuran	C_9H_8O	4265-25-2	FC	4	10	0.09	0.39
56.69	2-Ethyl-1-butene	$C_6H_6O_2$	620-02-0	Hc	12	13	1.96	0.69
57.26	Menthol	$C_{10}H_{20}O$	15356-70-4	Ter	85	97	19.86	25.17
57.86	Myrtenal	$C_{10}H_{14}O$	564-94-3	Ter	8	7	0.30	0.21
58.29	6-Methyl-3(2H)-pyridazinone	$C_5H_6N_2O$	13327-27-0	HHc	4	10	0.26	0.33
58.66	1,1,4,7-Tetramethylindan	$C_{13}H_{18}$	1078-04-2	Hc	35	43	3.34	3.57
58.76	α-Ionene	$C_{13}H_{18}$	475-03-6	Nor	8	10	0.83	0.62
59.66	Durene	$C_{10}H_{14}$	95-93-2	Ter	4	17	0.21	1.46
59.86	γ-Vinyl-γ-valerolactone	$C_7H_{10}O_2$	1073-11-6	FC	27	33	1.91	2.80
59.87	2,3,5,5,8,8-Hexamethyl-1,3,6-cyclooctatriene	$C_{14}H_{22}$	100016-19-7	Hc	0	0	-	-
59.87	2,5-Diisopropyl-p-xylene	$C_{14}H_{22}$	10375-96-9	BD	0	7	-	0.33

60.18	p-Menth-1(7)-en-2-one	$C_{10}H_{16}O$	15297-07-1	Ter	15	33	2.20	3.15
60.60	*α*-Cyclogeraniol	$C_{10}H_{18}O$	6627-74-3	Ter	4	0	0.22	-
61.06	1,5,8-Trimethyl-1,2,3,4-tetrahydronaphthalene	$C_{13}H_{18}$	21693-51-6	Nor	4	13	0.96	1.05
61.37	*α*-Methyl-cinnamaldehyde	$C_{10}H_{14}O$	585-34-2	Ald	8	53	0.40	5.53
61.42	4,7-Dimethylbenzofuran	$C_{10}H_{10}O$	28715-26-6	FC	27	43	1.45	6.14
61.49	1,1,6-Trimethyl-1,2,3,4-tetrahydronaphthalene	$C_{13}H_{18}$	30316-36-0	Nor	12	7	0.71	0.81
61.70	*m*-tert-Butylphenol	$C_{10}H_{14}O$	585-34-2	PC	19	7	1.74	1.72
62.28	6-tert-Butyltetralin	$C_{14}H_{20}$	42044-26-8	ND	15	0	1.03	-
62.31	Phellandral	$C_{10}H_{16}O$	21391-98-0	Ter	4	17	0.14	1.98
62.32	3,3,5,7-Tetramethyl-1-indanone	$C_{13}H_{16}O$	54789-23-0	Ket	23	17	1.71	1.59
62.47	Piperitone	$C_{10}H_{16}O$	89-81-6	Ter	31	37	4.08	5.01
62.55	2-Caranone	$C_{10}H_{16}O$	497-62-1	Ter	4	3	0.98	0.38
62.68	*p*-tert-Butylphenol	$C_{10}H_{14}O$	585-34-2	PC	46	67	4.03	12.79
63.00	1,2,3,4-tetramethyl-4-(1-methylethenyl)benzene	$C_{13}H_{18}$	61142-76-5	BD	12	17	0.96	1.63
63.23	D-Carvone	$C_{10}H_{14}O$	2244-16-8	Ter	62	100	19.90	48.01
63.79	1,1,6-Trimethyl-1,2-dihydronaphthalene	$C_{13}H_{16}$	30364-38-6	Nor	65	7	28.58	0.43
64.47	3-Methoxy-5-(trifluoromethyl)aniline	$C_8H_8F_3NO$	349-55-3	BD	81	93	11.61	17.15
64.62	*p*-Acetyltoluene	$C_9H_{10}O$	122-00-9	Ket	8	27	0.84	1.58
64.86	Salicylic acid, methyl ester	$C_8H_8O_3$	119-36-8	Est	38	40	5.82	5.63
64.92	2-Methyl-3-phenylpropanal	$C_{10}H_{12}O$	100013-18-7	Ald	8	20	0.28	4.26
65.12	1-(2,3,6-Trimethylphenyl)-3-buten-2-one	$C_{13}H_{16}O$	54789-45-6	Nor	8	10	1.15	1.63
65.26	1,4-Dimethoxy-2,3-dimethylbenzene	$C_{10}H_{14}O_2$	39021-83-5	BD	15	3	1.47	0.27
65.83	1-(3-Methylbutyl)-2,3,5-trimethylbenzene	$C_{14}H_{22}$	107997-60-4	BD	8	13	1.06	0.88
66.22	β-Damascenone	$C_{13}H_{18}O$	23726-93-4	Nor	77	63	7.52	10.64
66.48	3,5-Dimethyl benzaldehyde	$C_9H_{10}O$	5779-95-3	Ald	88	13	8.55	0.68
66.85	1,6,8-trimethyl-1,2,3,4-tetrahydronaphthalene-	$C_{13}H_{18}$	30316-36-0	Nor	38	50	2.10	4.13
67.83	2-methoxyphenol	$C_7H_8O_2$	90-05-1	PC	77	90	5.89	7.91
68.41	1,1-dimethylethyl-4-benzenethiol	$C_{10}H_{14}S$	2396-68-1	SC	12	23	0.77	2.47
68.68	1,3,3a,4,5,9b-hexahydro-2H-Benz[e]indol-2-one	$C_{12}H_{13}NO$	32940-67-3	Ket	4	0	0.90	-

69.05	1,2,3-Trimethylindene	$C_{12}H_{14}$	4773-83-5	Hc	8	40	0.24	2.93
69.36	2,3,6-trimethyl-4-methoxyphenol	$C_{10}H_{14}O_2$	53651-61-9	PC	31	3	2.43	0.18
69.84	1,1,6,8-Tetramethyl-1,2-dihydronaphthalene	$C_{14}H_{18}$	100018-66-3	ND	12	27	0.30	3.01
70.67	Acetate, (2,5,5,8a-tetramethyl-1,2,3,5,6,7,8,8a-octahydro-1-naphthalenyl) ester	$C_{16}H_{26}O_2$	101447-86-3	Est	46	83	2.79	10.14
70.95	Heptanoic acid	$C_7H_{14}O_2$	3665-80-3	CA	19	43	0.87	4.42
71.27	5,6-Dimethyltetralin	$C_{12}H_{16}$	20027-77-4	ND	15	23	0.68	2.41
71.38	N-Ethyl-4-nitroaniline	$C_8H_{10}N_2O_2$	3665-80-3	BD	42	57	2.86	4.60
71.90	2-Methoxy-p-cresol	$C_8H_{10}O_2$	93-51-6	PC	4	7	0.12	0.37
72.19	[(tetramethylcyclopropylidene)methyl]benzene	$C_{14}H_{18}$	85036-38-0	BD	15	7	1.18	0.30
72.51	2-Bromophenol	C_6H_5BrO	95-56-7	PC	77	97	4.41	10.04
72.93	2,4-Dimethyl Quinoline	$C_{11}H_{11}N$	1198-37-4	HHc	12	20	1.07	1.96
73.01	1,6-Dimethyl-4-(1-methylethyl)naphthalene	$C_{15}H_{18}$	483-78-3	Ses	0	10	-	1.27
73.22	4-Methoxy-2,3,6-trimethylphenol	$C_{10}H_{14}O_2$	53651-61-9	PC	35	50	1.67	2.41
73.38	2',6'-Dihydroxy-3'-methylacetophenone	$C_9H_{10}O_3$	29183-78-6	BD	31	10	1.61	0.71
73.64	Phenol	C_6H_6O	108-95-2	PC	100	97	22.30	20.32
74.11	3,5,8-trimethyl-1,2-dihydronaphthalene	$C_{13}H_{16}$	30316-18-8	Nor	0	10	-	0.74
74.26	1,4-Dimethoxy-2,3-dimethylbenzene	$C_{10}H_{14}O_2$	39021-83-5	HC	8	10	0.63	0.38
74.64	1,7-Dimethylnaphthalene	$C_{12}H_{12}$	575-37-1	ND	42	60	4.25	3.43
75.20	Octanoic Acid	$C_8H_{16}O_2$	124-07-2	CA	23	53	1.56	8.97
75.47	1-Methyl-2-phenylpiperidin-4-one	$C_{12}H_{15}NO$	91640-05-0	Ket	81	63	6.85	6.58
75.84	Azunol	$C_{15}H_{18}$	489-84-9	Ses	0	27	-	2.00
76.28	2,5,8-Trimethyl-1,4-dihydronaphthalene	$C_{13}H_{16}$	30316-19-9	Nor	38	43	4.29	6.41
76.62	*p*-Cresol	C_7H_8O	106-44-5	PC	100	100	71.61	55.20
77.14	1-Ethyl-3,5-dimethylbenzene	$C_{10}H_{14}$	934-74-7	Ter	4	0	0.14	-
77.45	Cumic alcohol	$C_{10}H_{14}O$	536-60-7	Ter	0	10	-	1.06
77.79	Paeonal	$C_9H_{10}O_3$	552-41-0	Ket	27	10	1.99	0.66
78.06	Eudalene	$C_{14}H_{16}$	490-65-3	ND	15	47	1.42	3.25
78.74	3,3,5,6-Tetramethyl-1-indanone	$C_{13}H_{16}O$	54789-22-9	Ket	81	87	8.68	8.18
79.48	Nonanoic acid	$C_9H_{18}O_2$	112-05-0	CA	50	47	4.57	4.24

80.39	2,3,3-Trimethylindanone	$C_{12}H_{14}O$	54484-71-8	Ket	8	20	0.14	1.64
80.45	Eugenol	$C_{10}H_{12}O_2$	97-53-0	PC	0	17	-	1.38
80.47	4,5,5-trimethyl-1,3-cyclopentadien-1-yl-benzene	$C_{14}H_{16}$	33930-85-7	BD	38	30	3.14	3.20
80.60	α,α,α-Trifluoro-p-cresol	$C_7H_5F_3O$	402-45-9	PC	15	10	0.69	0.70

Legend: Alc: Alcohols; Ald: Aldehydes; BD: Benzene Derivatives; CA: Carboxylic Acid; CTRL: control; Est: Esters; FC: Furanic Compounds; Hc: Hydrocarbons; HHc, Heterocyclic Hydrocarbons; Ket: Ketones; ND: Naphthalene Detivatives; Nor: Norisoprenoids; PC: Phenolic Componds; PCa: prostate cancer; SC: Sulfur Compounds; Ses: Sesquiterpenes; Ter: Terpenes; “-“: not identified.
